# Application of angiogenesis-related genes associated with immune infiltration in the molecular typing and diagnosis of acute myocardial infarction

**DOI:** 10.18632/aging.205936

**Published:** 2024-06-14

**Authors:** Guoqing Liu, Wang Liao, Xiangwen Lv, Miaomiao Zhu, Xingqing Long, Jian Xie

**Affiliations:** 1 Department of Cardiology, The First Affiliated Hospital of Guangxi Medical University, Nanning, Guangxi, China; 2 Department of Cardiology, The First People’s Hospital of Yulin, Yulin, Guangxi, China; 3 Department of Cardiology, The Second Affiliated Hospital of Guangxi Medical University, Nanning, Guangxi, China; 4 Guangxi Medical University, Nanning, Guangxi, China

**Keywords:** acute myocardial infarction, angiogenesis, predictive model, immune infiltration, molecular typing

## Abstract

Background: Angiogenesis has been discovered to be a critical factor in developing tumors and ischemic diseases. However, the role of angiogenesis-related genes (ARGs) in acute myocardial infarction (AMI) remains unclear.

Methods: The GSE66360 dataset was used as the training cohort, and the GSE48060 dataset was used as the external validation cohort. The random forest (RF) algorithm was used to identify the signature genes. Consensus clustering analysis was used to identify robust molecular clusters associated with angiogenesis. The ssGSEA was used to analyze the correlation between ARGs and immune cell infiltration. In addition, we constructed miRNA-gene, transcription factor network, and targeted drug network of signature genes. RT-qPCR was used to verify the expression levels of signature genes.

Results: Seven signature ARGs were identified based on the RF algorithm. Receiver operating characteristic curves confirmed the classification accuracy of the risk predictive model based on signature ARGs (area under the curve [AUC] = 0.9596 in the training cohort and AUC = 0.7773 in the external validation cohort). Subsequently, the ARG clusters were identified by consensus clustering. Cluster B had a more generalized high expression of ARGs and was significantly associated with immune infiltration. The miRNA and transcription factor network provided new ideas for finding potential upstream targets and biomarkers. Finally, the results of RT-qPCR were consistent with the bioinformatics analysis, further validating our results.

Conclusions: Angiogenesis is closely related to AMI, and characterizing the angiogenic features of patients with AMI can help to risk-stratify patients and provide personalized treatment.

## INTRODUCTION

Acute myocardial infarction (AMI) is usually caused by a ruptured plaque or thrombosis that blocks an artery and is characterized by decreased blood flow to the heart and cardiac muscle injury due to oxygen deficit, which can eventually lead to heart failure or even death [1]. Timely restoration of blood flow to ischemic and hypoxic tissues can reduce the occurrence of irreversible damage to functional cardiomyocytes, thereby improving the quality of survival and reducing mortality in patients with heart attacks [2]. Commonly used clinical measures to treat patients with myocardial infarction include thrombolytic therapy, reperfusion therapy, percutaneous coronary intervention (PCI), and coronary artery bypass grafting (CABG) [3]. Although medications and interventions have significantly improved survival rates in patients with heart attacks, patients with AMI are at high risk for complications such as ischemia and reperfusion injury, which are the leading causes of chronic heart failure [4]. Therefore, it is necessary to explore further the mechanisms of occurrence and new treatments to preserve myocardial function in AMI and avoid the onset and progression of heart failure.

Promoting angiogenesis is a potential protective strategy to prevent myocardial injury and improve the prognosis of myocardial infarction. Angiogenesis consists of the sprouting of endothelial cells from preexisting vessels that are primarily dependent on endothelial cells to promote the formation of new capillaries and collateral arteries that deliver nutrients to the infarcted region and also provide energy to myofibroblasts to prevent long-term left ventricular remodeling and chronic heart failure [5]. Angiogenesis is regulated by multiple signaling pathways, with triggers including hypoxia, intercellular communication, angiogenic factors, and inflammation-driven [6–9]. The hypoxia-inducible factors (HIF), expressed in cardiomyocytes, endothelial, and inflammatory cells, are one of the main factors driving angiogenesis by hypoxia. The exosomes overexpressing HIF-1α can regulate the expression of vascular endothelial growth factor (VEGF), angiopoietin 1 (Ang-1), and other encoded angiogenic factors to induce angiogenesis [10, 11]. VEGF is a pro-angiogenic factor upregulated in ischemic heart disease, regulating angiogenesis by binding and interacting with VEGF receptors (VEGFRs) to facilitate myocardial repair [10]. Grellier et al. demonstrated the role of intercellular communication on primary angiogenesis by establishing a monolayer cell model of human umbilical vein endothelial cells [11]. The newly formed dense capillary network provides a pathway for inflammatory cells, promotes metabolism and energy exchange in the infarcted region, and protects normal cardiomyocyte function [12].

It has been demonstrated that immune infiltration is a critical procedure in the angiogenesis and repair phases of AMI. Monocytes/macrophages are the main immune regulators of angiogenesis [13]. By establishing a mouse model of AMI, Wu et al. found that monocytes and macrophages secreted pro-angiogenic factors other than VEGFA, such as growth factors and nuclear chemokines, which interacted with neighboring cells in a paracrine manner to promote angiogenesis after myocardial injury [14]. Neutrophils are divided into two main subtypes, N1 and N2, of which N2 is a critical player in tumor angiogenesis and metastasis in mice and humans [15]. These findings suggest that immune infiltration is a new direction to prevent infarct progression and that exploring immune infiltration and its relationship with angiogenesis can help analyze the pathogenesis of AMI and provide therapeutic strategies.

Clinical research on angiogenesis in cardiovascular disease has progressed rapidly in recent years, but its mechanism of action in AMI is still unclear. In this study, we screened signature angiogenesis-related genes (ARGs) in AMI using machine learning algorithms. In addition, we performed an immune infiltration analysis of ARGs to explore the association of the immune microenvironment and angiogenesis, which provides a risk basis for clinical decision-making and some rationale for new therapeutic approaches for AMI.

## MATERIALS AND METHODS

### Data sources and processing

The GSE66360 and GSE48060 datasets containing AMI and normal samples were collected from the Gene Expression Omnibus (GEO) (https://www.ncbi.nlm.nih.gov/geo/) database. These two datasets were derived from the chip-based platform GPL570 [HG-U133_Plus_2] Affymetrix Human Genome U133 Plus 2.0 Array. The GSE66360 dataset (circulating endothelial cell samples from 49 AMI and 50 normal samples of humans) served as the training cohort, while the GSE48060 (serum samples from 31 AMI and 21 normal samples of humans) dataset served as the external validation cohort. 36 ARGs were downloaded from the MSigDB Team (Hallmark Gene set). In addition, we used the Perl software to annotate the dataset and convert the data into a gene expression profile for subsequent analysis.

### Differential expression and correlation analysis

Differentially expressed ARGs in control and AMI samples were analyzed by the “limma” package in R. Samples with a *P* < 0.05 and |log2-fold change (FC)| ≥ 1 were considered as the threshold points for differentially expressed genes (DEGs). Furthermore, we performed a correlation analysis to explore co-expression characteristics among ARGs.

### Construction and selection of algorithms

In this study, we constructed and compared models based on two machine learning algorithms, including random forest (RF) and support vector machine (SVM). RF incorporates a bagging algorithm and randomized feature algorithm, which can be effectively self-supervised and has more significant advantages over other machine learning methods [16]. The SVM is constructed based on the statistical learning principle of risk minimization through structure and has been widely used for genome classification or subtyping in recent years [17]. We effectively evaluated both algorithms by box line plots of residuals, inverse distribution plots of residuals, receiver operating characteristic (ROC) curves, and random forest tree plots.

### Establishment of the nomogram

We drew a nomogram to visualize the risk predictive model based on the signature genes to predict the occurrence of AMI. In the nomogram, the corresponding scores were assigned to the impact levels of each of the signature genes, and the score values of each gene were summed to obtain the total score, which in turn predicted the risk of disease occurrence by function transformation. In addition, decision curve analysis (DCA), calibration, clinical impact curves (CIC), and ROC curves were used to evaluate the accuracy of the risk predictive model.

### Molecular typing and immune infiltration analysis of ARGs

We used the “ConsensusClusterPlus” package in R to perform consensus clustering to identify the ARG pattern. Subsequently, the groupings were validated by principal component analysis (PCA). Based on single sample gene set enrichment analysis (ssGSEA), we analyzed the correlation between ARGs and immune cell infiltration. ARGs associated with immune infiltration were selected for the subsequent study.

### Identification and functional analysis of DEGs

The “limma” package was performed to identify DEGs between two ARG subclusters. And |log2-FC| ≥ 1 and *P* < 0.05 were selected as the criteria. The “clusterProfiler”, “org.Hs.eg.db”, “GOplot”, and “enrichplot” packages were applied to perform the enrichment analyses of Gene Ontology (GO) and Kyoto Encyclopedia of Genes and Genomes (KEGG) pathways, with a *P*-value filter < 0.05 item included. We plotted correlation graphs with the “corrplot” package to obtain bubble and string plots for the GO enrichment circle and KEGG enrichment analysis. We examined the potential biological functions of DEGs in terms of molecular functions (MF), biological processes (BP), and cellular components (CC).

### ARG scores in two different modes

We mainly used PCA for ARG scores in the ARG cluster and ARG gene cluster and thus verified the accuracy of ARG pattern grouping. Subsequently, the “ggalluvial” package in R was used to analyze the association between the ARG cluster, gene cluster, and ARG scores.

### Construction of targeted drug network

The search for targeted drugs for signature genes was based on the DGIdb database (http://dgidb.genome.wustl.edu/) [18], and the gene-drug network was drawn and visualized by the Cytospace software (version 3.7.2).

### Construction of miRNA network and transcription factor network

Based on Network Analyst (https://www.networkanalyst.ca), we predicted the miRNA and transcription factors of signature genes. We then used Cytospace software (version 3.7.2) for visualization.

### Cell culture and construction of the model

This study derived the AC16 cells (human cardiomyocyte cells) from the cell bank (Procell; Wuhan, China). We cultured AC16 cells in Dulbecco’s modified Eagle’s medium (DMEM) (Gibco, Thermo Fisher Scientific, Waltham, MA, USA), which included 10% fetal bovine serum (FBS), 100 U/mL penicillin, and 100 mg/ml streptomycin (Beijing Solarbio Science and Technology Co., Ltd., Beijing, China), at a constant temperature of 37° C and the status of 5% CO_2_.

A suitable density of cells in the logarithmic growth phase was seeded into a medium supplemented with 10% FBS and incubated overnight. AC16 cells were split into the control group (Con group) and the oxygen-glucose deprivation treatment group (OGD group).

According to the relevant experimental conditions, the AC16 cells were evenly dispersed and cultured in an incubator for 24 hours. We placed the plates in a hypoxic chamber with an inlet and outlet. Next, the outlet port valve was opened, and the anoxic chamber was filled with the mixed gas containing 5% CO_2_ and 95% N_2_ through the inlet port. Shut the air inlet and outlet simultaneously to create an oxygen-free closed chamber, which would be put into an incubator at 37° C. The cells were taken out of the hypoxic chamber after 6 hours and needed additional tests by the follow-up experiments.

### The analysis of reverse transcription quantitative polymerase chain reaction (RT-qPCR)

The DMEM solution was supplemented with 10% FBS, where the cells were developed. When the cell confluence rate reached 70% to 80%, we cultivated all cells in an incubator with a constant temperature of 37° C and the status of 5% CO_2_. Trypsin solution was employed to digest and subculture these cells, and then cells in the logarithmic growth stage would be removed for further RT-qPCR.

The RNA in each group was extracted by TRIzol kit reagents (Invitrogen, Waltham, MA, USA) and was reverse transcribed into cDNA by QuanTiect reverse transcription kit (Qiagen, Hilden, Germany). RT-qPCR was performed with SYBR Green (Takara, Shiga, Japan) CFX96 Real-time PCR system (Bio-Rad Laboratories, Hercules, CA, USA). GAPDH was used to normalize expression levels. Table 1 contains a list of primer sequences. The 2^-ΔΔCT^ approach was used for data analysis.

**Table 1:** Primers used for RT-qPCR.

**Gene**	**Primers**	**Sequence (5’–3’)**
THBD	Forward	GGAGACAACAACACCAGCTATA
	Reverse	GGAAGTGGAACTCGCAGA
VCAN	Forward	ACTGAAACTTCCTACGTATGCA
	Reverse	CTCACAAAGTGCACCAACATAA
CCND2	Forward	TTTAAGTTTGCCATGTACCCAC
	Reverse	ACGTCTGTGTTGGTGATCTTAG
VEGFA	Forward	ATCGAGTACATCTTCAAGCCAT
	Reverse	GTGAGGTTTGATCCGCATAATC
JAG1	Forward	ATTACCAGGATAACTGTGCGAA
	Reverse	CAAATGTGCTCCGTAGTAAGAC
POSTN	Forward	CACCAATGAGGCTTTTGAGAAA
	Reverse	GACTGCTCCTCCCATAATAGAC
PGLYRP1	Forward	CACTCAGGTCACTTATGGAACC
	Reverse	GTGTCCTTTGAGCACATAGTTG
GAPDH	Forward	GGAGTCCACTGGCGTCTTCA
	Reverse	GTCATGAGTCCTTCCACGATACC

### Statistical analysis

Data processing and collation via Perl software (Version 5.18.2). Data analysis was mainly implemented by R software (version 4.1.2). Student’s t-test or Wilcoxon’s rank sum test was used to detect the significant difference between the two independent groups. It was considered statistically significant with *P* < 0.05.

## RESULTS

### The landscape of the ARGs in AMI

**Figure 1:**
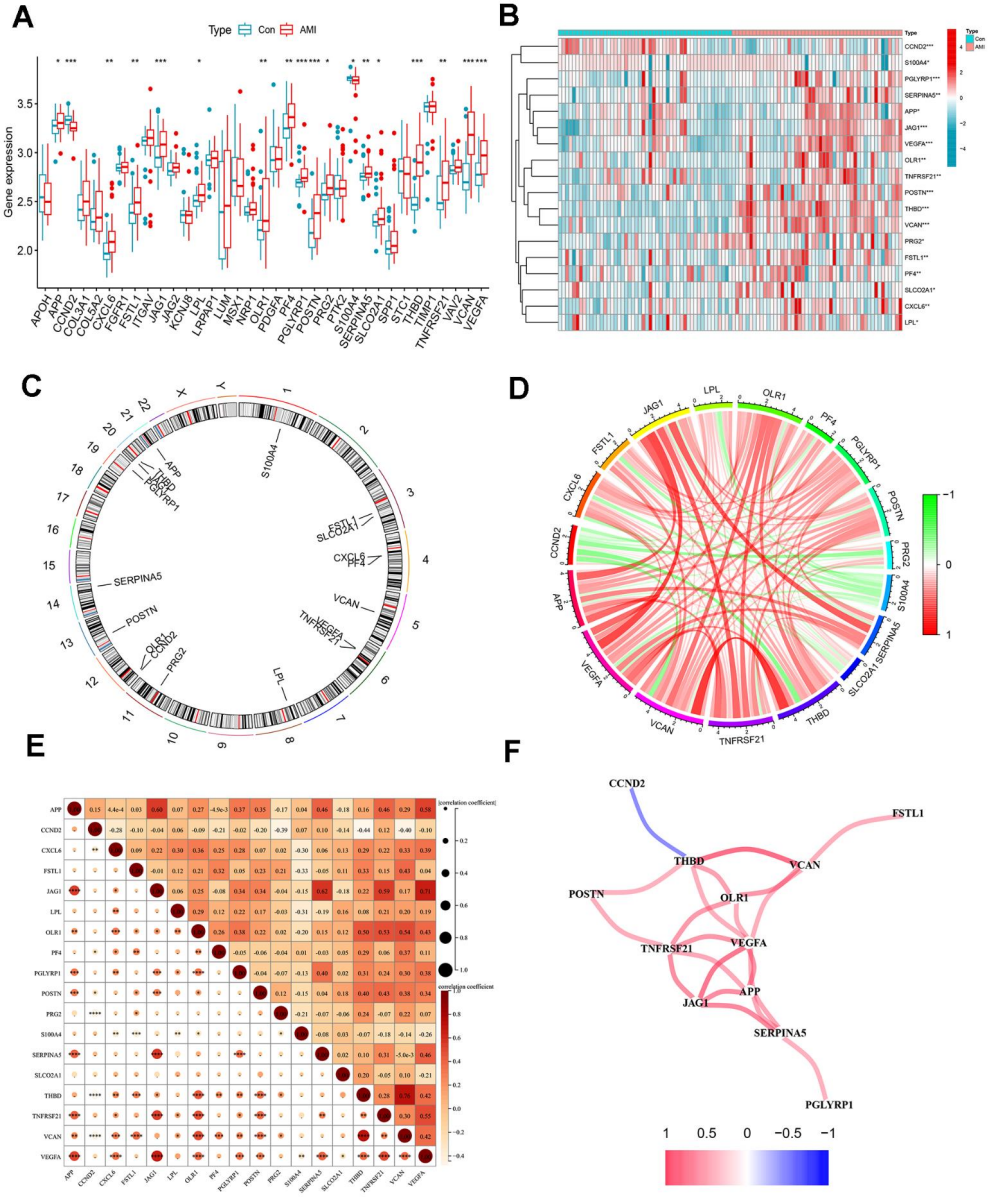
**Differentially expressed ARGs in AMI and correlation analysis.** (**A**) Boxplots for differentially expressed ARGs between healthy and AMI groups. (**B**) Heat map of differential expression of the 18 ARGs. (**C**) Circos plot of 18 ARGs: showed the distribution of each gene on the chromosomes. (**D**) Chordal graph of 18 ARGs correlations. (**E**) Correlation heat map. (**F**) Interaction network analysis diagram: connecting lines represented interaction relationships. *P < 0.05; **P < 0.01; ***P < 0.001.

Differential expression analysis between healthy individuals and patients with AMI was performed, and 18 differentially expressed ARGs were selected (Figure 1A). Among them, the expression of most genes included APP, CXCL6, FSTL1, JAG2, LPL, OLR1, PF4, PGLYRP1, POSTN, PRG2, SERPINA5, SLCO2A1, THBD, TNFRSF21, VCAN, and VEGFA were significantly upregulated in AMI samples, while S100A4 and CCND2 were downregulated in AMI samples (Figure 1B). The location of the 18 differentially expressed ARGs on the various chromosomes was displayed in Figure 1C.

### Correlation analysis of differentially expressed ARGs

Linear correlation analysis was adopted to explore the association between each differentially expressed ARG (Figure 1D, 1E). Figure 1F showed a positive correlation network among THBD, VCAN, OLR1, TNFRSF21, VEGFA, JAG1, APP, and SERPINA5. VEGFA was positively correlated with THBD, JAG1, and TNFRSF21. The most substantial positive correlation relationship was found between VCAN and THBD. THBD was also negatively related to CCND2. In addition, the positive correlation between APP and JAG1, JAG1, and SERPINA5 was higher, with correlation coefficients over 0.6.

### Selection of algorithms and construction of predictive model

**Figure 2:**
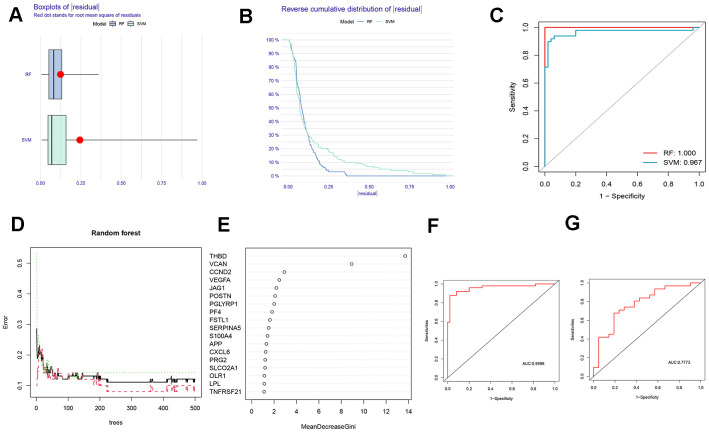
**Comparison and selection of random forest (RF) and support vector machines (SVM).** (**A**) Residual Boxplot of RF and SVM. (**B**) Reverse cumulative distribution of residual. (**C**) Receiver operating characteristic (ROC) curve of RF and SVM. The AUC of RF and SVM were 1.000 and 0,967, respectively. (**D**) RF prediction error curves based on a 10-fold cross-validation curve. Treat groups (red line), control groups (green line), and overall samples (black line). (**E**) The scoring plot of each gene. The higher the score, the more important. (**F**) The ROC curve of the logistic regression model constructed in AMI based on hub HRGs identified by RF algorithm in the training cohort. (**G**) The ROC curve of the logistic regression model constructed in AMI in the testing cohort.

Based on boxplots and reverse cumulative distribution plots of residuals (Figure 2A, 2B), we found that the RF algorithm had lower residuals than the SVM algorithm, indicating its higher accuracy for predicting the occurrence of AMI. Meanwhile, the ROC curve was plotted (Figure 2C). It further supported the conclusion that the RF algorithm was more appropriate than SVM because of its larger area under the curve (AUC).

A 10-fold cross-validation curve was drawn to obtain a more reasonable and accurate evaluation of the model, exhibiting the error levels of the treatment groups, the control groups, and all samples (Figure 2D). The mean decrease Gini represents the importance of each gene, and the genes with a Gini index greater than 2 were selected for subsequent analysis (Figure 2E).

The above findings revealed that the RF algorithm could be implemented as a valid model to forecast the risk of AMI. Based on the 7 signature genes, a binary logistic regression model was used to predict the risk of AMI. The ROC curve was exhibited in Figure 2F, 2G. The training and the external validation cohorts for the classification model demonstrated satisfactory discrimination performance (AUC = 0.9596 and 0.7773, respectively).

### Visualization and validation of the risk predictive model

**Figure 3:**
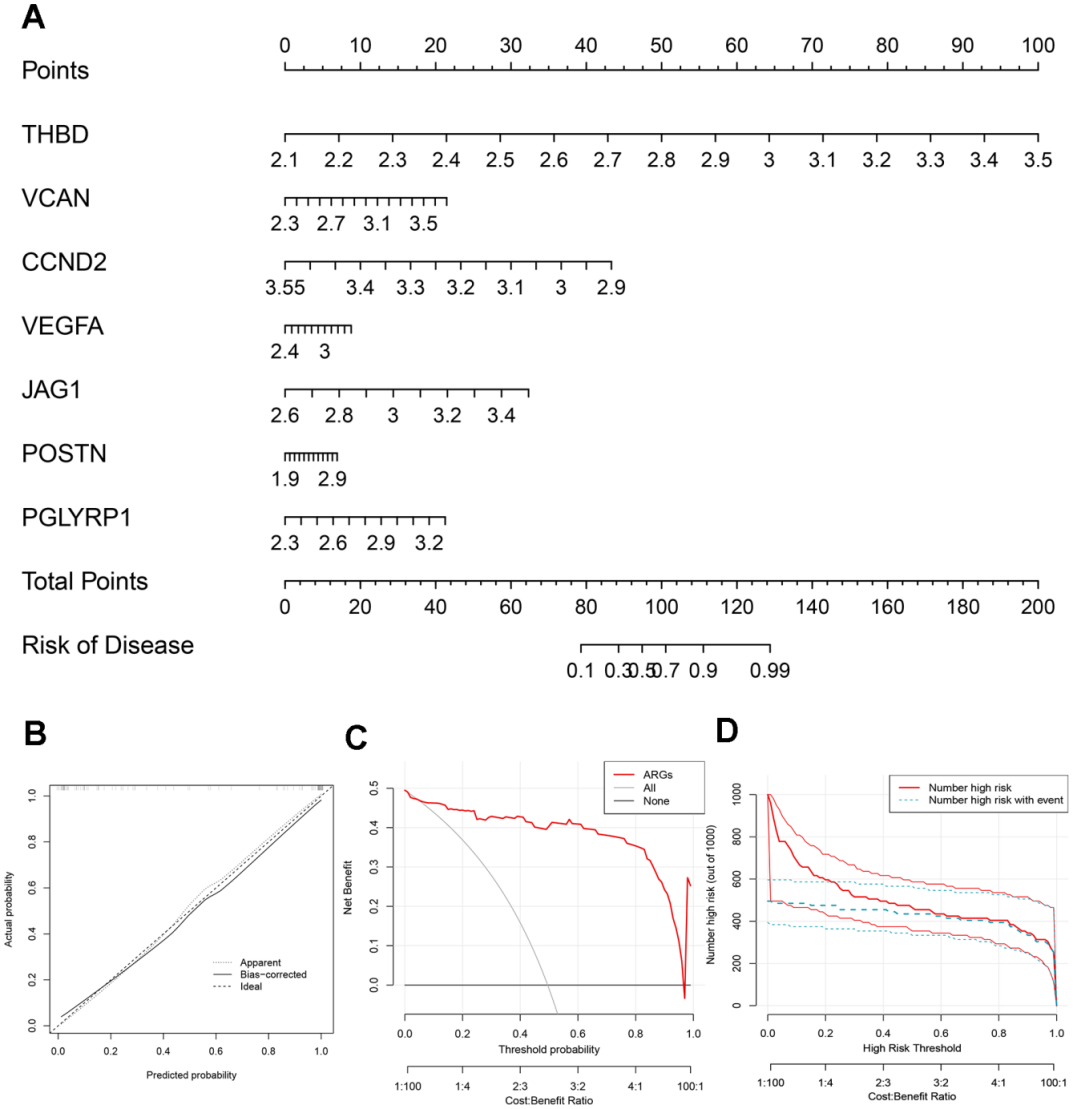
**Construction of the nomogram.** (**A**) The nomogram based on 7 signature ARGs. (**B**) The calibration curve: assessed prediction accuracy. (**C**) Decision curve analysis (DCA) evaluated the clinical utility of the predictive model. (**D**) The clinical impact curve (CIC) proved the clinical value of the predictive model.

A nomogram was plotted to visualize the predictive models of AMI based on the sum of each gene expression score (Figure 3A). 7 genes included THBD, VCAN, CCND2, VEGFA, JAG1, POSTN and PGLYRP1 were involved in the model. The bias-corrected line was close to the ideal dotted line in Figure 3B, illustrating the model’s good prediction ability. Besides, DCA, in which the curve of ARGs deviated from two extreme ones, was performed, indicating the model’s great clinical utility (Figure 3C). The CIC proved the availability of a nomogram by comparing the number of predicted and actual patients under different probability thresholds (Figure 3D).

### Relationship between 7 signature genes

**Figure 4:**
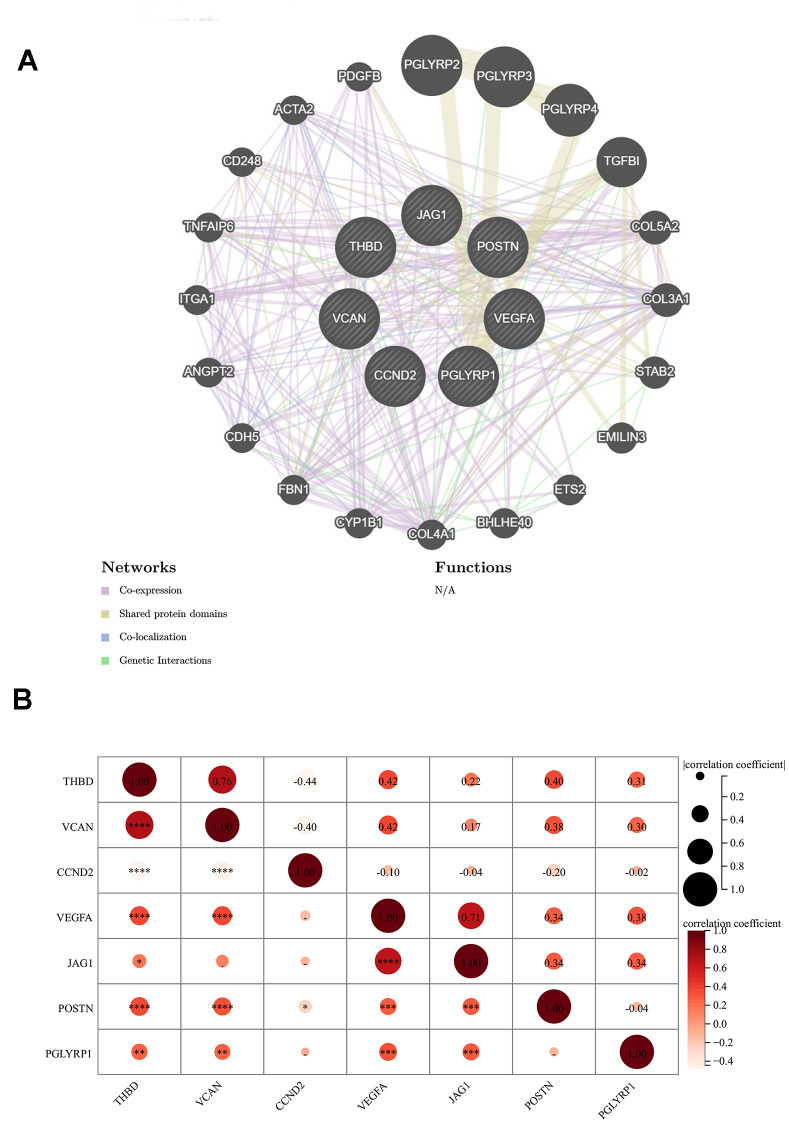
**Relationship between 7 signature genes.** (**A**) Genetics interaction networks of 7 signature ARGs based on GeneMANIA database. (**B**) Correlation analysis among 7 signature ARGs. *P < 0.05; **P < 0.01; ***P < 0.001.

Figure 4A revealed the network of the relationships between the 7 signature genes and the proteins predicted by the GeneMANIA database. We found that PGLYRP1, PGLYRP2, PGLYRP3, and PGLYRP4 all belong to the peptidoglycan recognition protein family, sharing protein domains, and have a strong relationship with each other, but without a co-expression relationship. TGFBI and POSTN were another pair with a similar structure. The co-expression relationship between the individual proteins was universal.

The result of the correlation analysis of each significant gene was shown in Figure 4B. A significant positive correlation between THBD and VCAN could not be ignored, while CCND2 was respectively negatively correlated with them. The correlation coefficient between any two genes in VEGFA, POSTN, THBD, and VCAN was about 0.4.

### Identification of ARG patterns

**Figure 5:**
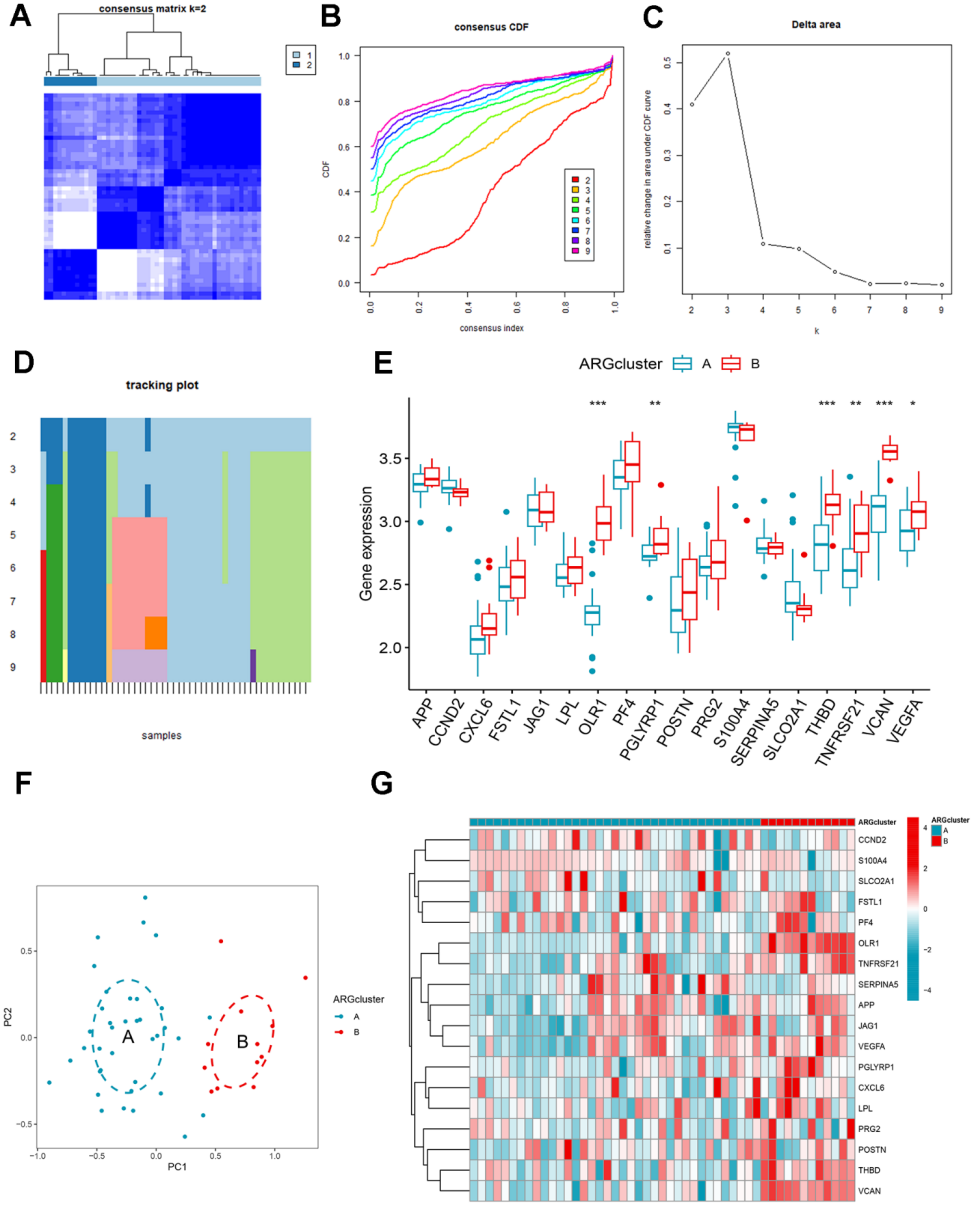
**Identification and gene expression analysis of ARG patterns.** (**A**) Consensus matrix with k=2. (**B**) Cumulative distribution function (CDF) of consensus clustering. (**C**) Delta area plot of consensus clustering. (**D**) Tracking plot. The abscissa represented different samples, and various color blocks indicated subtypes. (**E**) The expression of 18 ARGs in different clusters. (**F**) Principal component analysis (PCA). (**G**) Differential expression heat map of 18 ARGs.

To further explore the connection between ARGs and AMI, we performed a consensus clustering of angiogenic patterns based on 18 differentially expressed ARGs. Combining the result of Figure 5A–5D, two ARG patterns were separated based on 7 signature ARGs. Among the differentially expressed genes, the expression of OLR1, PGLYRP1, THBD, TNFRSF21, VCAN, and VEGFA was higher in cluster B (Figure 5E, 5G). The PCA showed that 7 ARGs could distinguish two patterns (Figure 5F).

**Figure 6:**
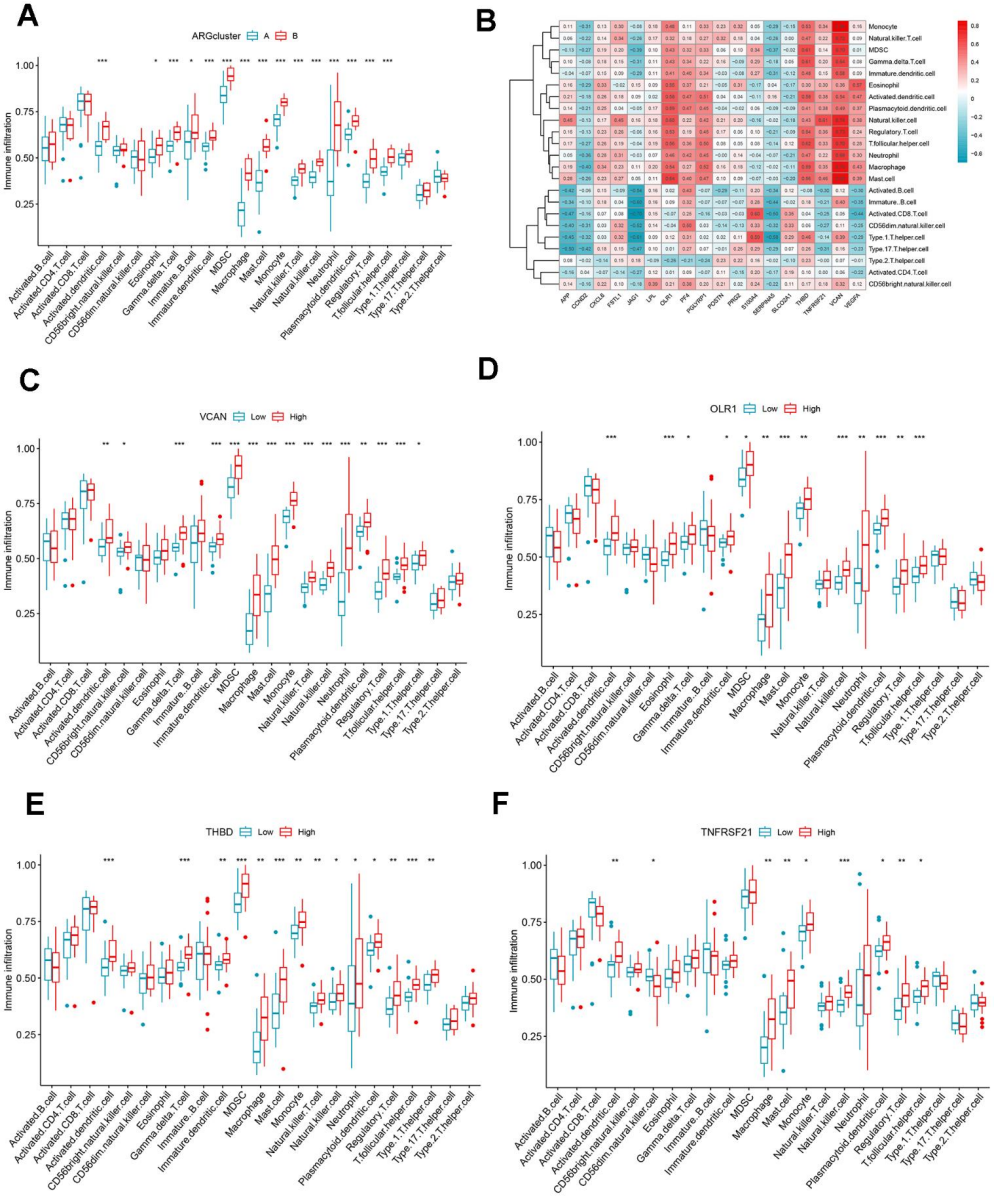
**Immune cell infiltration analysis.** (**A**) The correlation between infiltrating immune cells and ARG clusters. (**B**) Heat map of ARGs and immune cell relationships. (**C**–**F**) Comparison of immune cell abundance between the high and low expression groups of VCAN, OLR1, THBD, and TNFRSF21. *P < 0.05; **P < 0.01; ***P < 0.001.

The differences in the composition of diverse immune cells in the two ARG patterns were shown in Figure 6A. The immune cells with significant differences in clusters A and B mainly included activated dendritic cells, immature dendritic cells, macrophages, mast cells, monocytes, neutrophils, regulatory T cells, natural killer cells, and eosinophils. All differentially expressed immune cells were higher in cluster B. Then, we assess the relationship between ARGs and immune cells (Figure 6B), CCND2, JAG1, and SERPINA5 were negatively correlated with immune cells, while OLR1, PF4, PGLYRP1, S100A4, THBD, TNFRSF21, VCAN, and VEGFA were directly proportional to many immune cells. We split the expression levels of 18 ARGs into high or low groups to evaluate the diverse immune cell infiltration more thoroughly. Boxplots displayed that almost all discrepant immune cells involved activated dendritic cells, macrophages, mast cells, monocytes, natural killer cells, plasmacytoid dendritic cells, regulatory T cells, T follicular helper cells infiltrated in high expression of VCAN, OLR1, THBD, and TNFRSF21 apart from CD56^dim^ natural killer cell which infiltrated more frequently in low expression group of TNFRSF21 (Figure 6C–6F).

### Functional pathway analysis and identification based on ARG gene patterns

**Figure 7:**
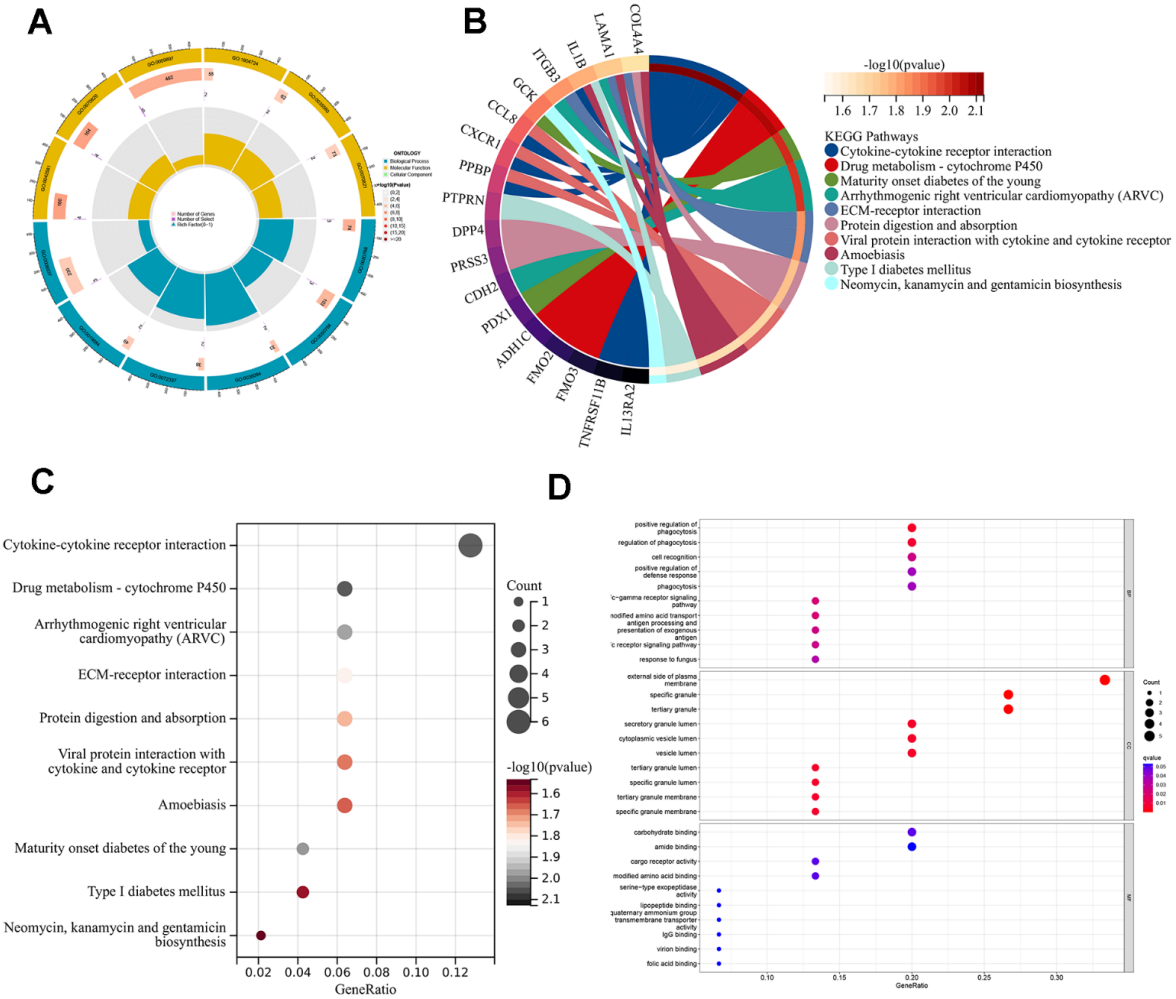
**Enrichment analyses of DEGs in different ARG pantens.** (**A**) GO enrichment analysis circle diagram. (**B**) Chordal graph of KEGG pathways and 18 genes. (**C**) A bubble chart for KEGG analysis. (**D**) A bubble chart for GO analysis.

To better understand the feasible mechanisms of DEGs in AMI, we performed GO and KEGG analysis in two different ARG modes. GO results depicted that DEGs mainly aggregated in terms of BP and MF. These differential genes were mainly enriched in the positive regulation of phagocytosis, Fc-gamma receptor signaling pathway, cell recognition, external side of the plasma membrane, specific granule, and tertiary granule (Figure 7A, 7D). KEGG results showed that the DEGs were mainly enriched in cytokine-cytokine receptor interaction, drug metabolism-cytochrome P450, and arrhythmogenic right ventricular cardiomyopathy (ARVC) (Figure 7B, 7C).

**Figure 8:**
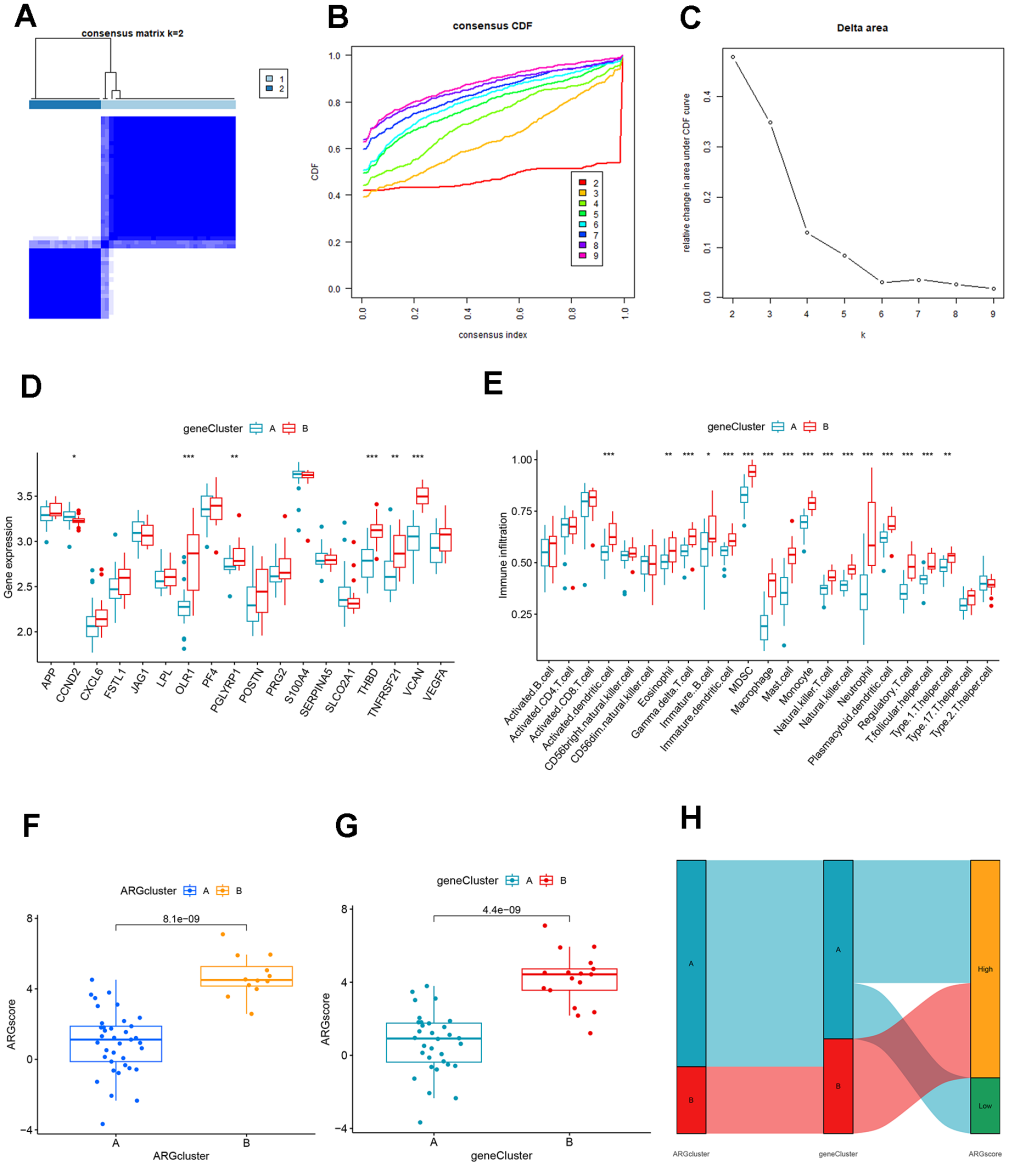
**Identification and comparison of different ARG gene patterns.** (**A**) Consensus matrix of DEGs with k=2. (**B**) CDF of consensus clustering. (**C**) Delta area plot of consensus clustering. (**D**) Differential expression analysis of clusters A and B genes based on ARG gene pattern. (**E**) Association of DEGs with immune cell infiltration in clusters A and B based on ARG gene pattern. (**F**) ARG score analysis between different clusters based on the ARG model. (**G**) ARG score analysis between different clusters based on the ARG gene model. (**H**) The association between ARG patterns, gene cluster, and ARG score was shown on the Sankey diagram.

We used the consensus clustering method to divide the patients with AMI into different genetic models and found that the results were consistent with the ARG mode (Figure 8A–8C). The grouping accuracy was further supported by the findings of differential expression analysis and immune cell infiltration in gene patterns, which were comparable to those of the ARG model (Figure 8D, 8E). Moreover, we also used PCA to calculate the scores between different samples of the ARG model as well as the gene model, and the results showed higher scores for clusters B or gene clusters B (Figure 8F, 8G). Furthermore, the Sankey diagram showed the relationship between the ARG, the genes, and the ARG score (Figure 8H).

### Construction of miRNA network map and transcription factor network map based on signature genes

**Figure 9:**
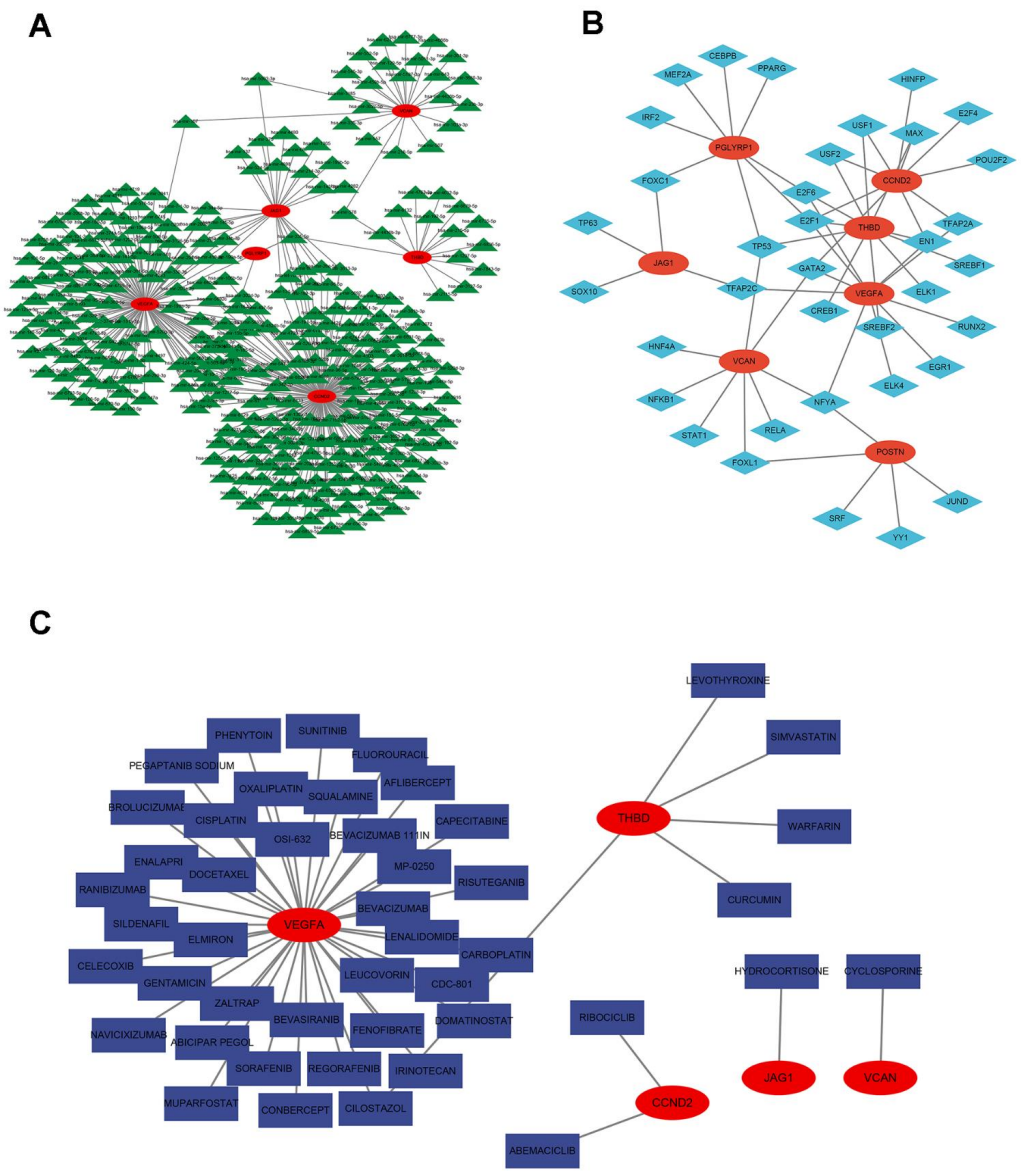
**Regulatory network of 7 signature ARGs.** (**A**) Network of miRNA-gene. (**B**) Network of transcription factor-gene. (**C**) Analysis of targeted drug prediction of signature ARGs.

The miRNA network map included 326 nodes, including 6 signature genes and 320 miRNAs. We found the highest number of identical target miRNAs responsible for regulating VEGFA with CCND2 and the lowest number of miRNAs responsible for regulating PGLYRP1. The network diagram showed that some miRNAs were associated with two signature gene expressions. MiR-335-5p target mRNA was PGLYRP1 and THBD; miR-5003-3p target mRNA were VCAN and JAG1; has-miR-107 target mRNA were VCAN and VEGFA (Figure 9A and Supplementary Table 1). Figure 9B showed the seven signature genes with transcription factors, and it showed that transcription factor FOXC1 acted on PGLYRP1 and JAG1; transcription factor TFAP2C acted on THBD, VCAN, and PGLYRP1; transcription factor NFYA acted on VEGFA, VCAN, and POSTN; transcription factors USF1 and USF2 both acted on CCND2 and THBD (Supplementary Table 1).

### Targeted drug prediction based on signature genes

By visualizing the targeting drugs for the signature genes, we found the targeting drugs closely related to VEGFA, including Elmiron, docetaxel, MP-0250, bevacizumab, bevasiranib, celecoxib, sildenafil, phenytoin, and sunitinib. Targeted drugs closely related to THBD include levothyroxine, simvastatin, and warfarin. We also found the target drug abemaciclib for CCND2 and cyclosporine for VCAN (Figure 9C and Supplementary Table 1).

### Validation of the expressions of signature genes

**Figure 10:**
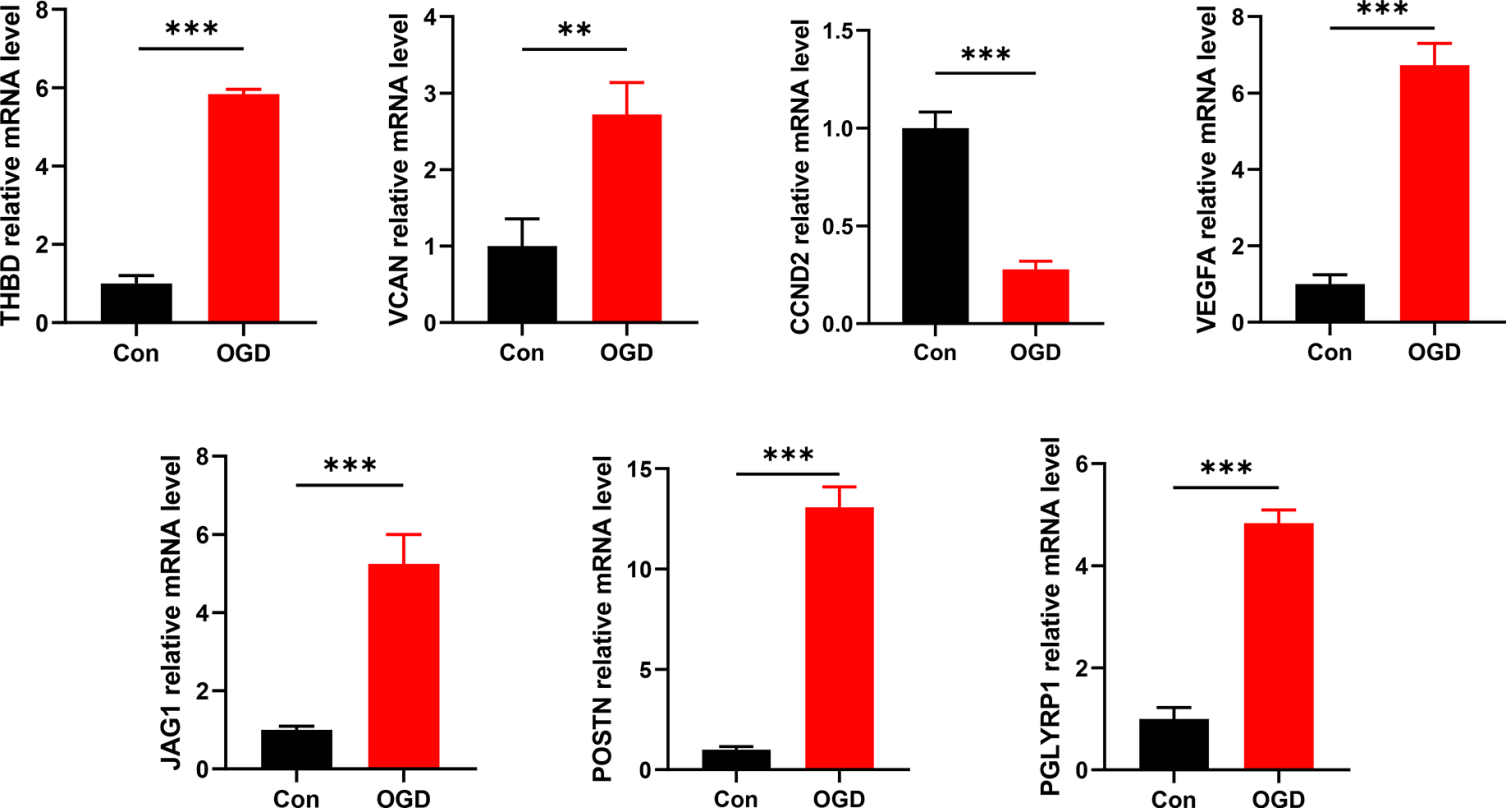
**Detection of mRNA expression levels of 7 signature ARGs by RT-qPCR.** *P < 0.05; **P < 0.01; ***P < 0.001.

In contrast to control groups, the expression of THBD, VCAN, VEGFA, JAG1, POSTN, and PGLYRP1 in OGD groups was significantly increased, while the expression of CCND2 in OGD groups was significantly decreased (all *P* < 0.05) (Figure 10). It was consistent with the results of the bioinformatics analysis and further validated our conclusions.

## DISCUSSION

Ischemia and hypoxia can initiate compensatory angiogenesis, which plays a vital role in injury repair after AMI [19]. In this study, we screened 7 signature ARGs from 18 differentially expressed ARGs between the control and AMI groups and constructed a risk predictive model of AMI. Moreover, we identified 2 ARG patterns based on 18 differentially expressed ARGs and explored their association with immune infiltration level and risk score of AMI, guiding the early prevention and treatment in patients in different ARG clusters. We constructed the miRNA and transcription factor network, providing new ideas for finding potential upstream targets and biomarkers. In addition, we explored possible target gene drugs that characterize ARGs, providing evidence for targeted therapy.

Precision medicine optimises outcomes by minimizing adverse events by adapting treatments to individual patient characteristics (clinically identified risk factors, biomarkers, pharmacogenomics, etc.) [20]. However, risk stratification of patients based on potential risk factors and risk evaluation systems (such as the Global Registry of Acute Coronary Events [GRACE] and the Thrombolysis in Myocardial Infarction [TIMI] risk scores) no longer fully meets the needs of clinical practice [21]. Combining traditional models with genetic information, machine learning algorithms, and artificial intelligence techniques can help improve the accuracy of risk assessment. In this context, we further explored the potential role of angiogenesis in AMI and identified signature ARGs that may serve as potential biomarkers by machine learning methods.

Studies have shown that promoting angiogenesis at the border of the infarct is essential for cardiac repair. Endothelial progenitor cells (EPCs) can induce cardiovascular formation in the ischemic zone through the differentiation of endothelial colony-forming cells (ECFCs) into endothelial cells and early outgrowth cells (EOCs) that secrete pro-angiogenic factors such as VEGF and chemokines [22]. VEGFA is the most important factor inducing angiogenesis, which is associated with VEGFR-1 and VEGFR-2 receptors on the surface of endothelial cells to regulate their proliferation and migration increase vascular permeability, and promote the activation of inflammatory factors as well [23], initiating angiogenesis on the fourth to tenth day after AMI. CCND2 belongs to the highly conserved cyclin family and can bind to Cyclin-dependent kinases 4 (CDK4) to regulate the G1 to S phase in the cell cycle [24]. Research has demonstrated that Human-induced pluripotent stem cells with upregulated CCND2 can increase the number of VEGFR-positive cells and facilitate angiogenesis in the border zone of AMI [25]. It is proven that CCND2 can promote myocardial repair in AMI Amice and swine, being a potential measure for AMI therapy [25, 26]. Another benefit of AMI treatment is THBD. It has been regarded as an anticoagulant receptor distributed on the luminal surface of all vascular endothelial cells [27]. A study found that THBD secreted from vascular endothelial cells promotes EPC growth and reduces apoptosis [28, 29]. However, there needs to be more research on the relationship between these 7 ARGs and AMI. Thus, the results of this study will act as a theoretical basis for subsequent research on ARGs and AMI.

This study divided the AMI sample into two ARG subclusters based on consensus clustering. Between clusters A and B, there were significant differences in 15 different types of immune cell infiltration, all of which were higher in cluster B, including macrophage, monocyte, neutrophil, natural killer T cell, dendritic cell, and so on. It may suggest that group B may be more sensitive to immunotherapy. Persistent non-resolving inflammation is one of the main factors in poor prognosis of AMI, and macrophages and neutrophils with phagocytic effect have a vital role. Previous studies have demonstrated that monocytes are recruited to the infarct site 2 hours after infarction, and cardiac resident macrophages die and are cleared within 24 hours and replaced by monocyte-derived macrophages. In the first 3 days, they exhibit inflammation subtypes (M1) initially, then transform into anti-inflammatory phenotypes (M2) to regulate the inflammatory cascade [30]. Neutrophils also have a similar process; three days after AMI, pro-inflammatory neutrophils N1, a crucial factor in left ventricular infarct wall thinning, exhibit an anti-inflammatory phenotype and undergo apoptosis [30, 31]. They selectively degranulate during AMI depending on their protein profile and the extracellular matrix (ECM) environment [32]. Neutrophil gelatinase-associated lipocalin (NGAL) can polarize macrophages towards the M2 phenotype [33]. In general, the resolution of inflammation is a complex process in which multiple immune cells interact and influence each other. The effect of different immune cell subtypes should be considered when treating myocardial remodeling in AMI.

We further explore the immune infiltration condition in high and low-expression groups of VCAN, OLR1, THBD, and TNFRSF21. All of them are positive correlation ARGs. Some kinds of dendritic cells, macrophages, mast cells, monocytes, natural killer cells, regulatory T cells, and T follicular helper cells significantly differed in their high expression group. Studies have shown that DCs can regulate post-infarction repair by promoting the release of inflammatory factors and activating fibroblasts; mast cells have a bidirectional effect on myocardial inflammation and fibrosis; Treg cells have anti-inflammatory properties while triggering fibrosis to promote repair [34]. In addition, natural killer cells may be related to monocytes via the cytokine axis T-bet/INF-γ/IL-12, promoting one another’s activity and stimulating inflammation in the infarction zone [35]. In conclusion, immune cells are vital participants in AMI, impacting immunotherapy. Abnormal activation of angiogenesis may promote the degree of immune cell infiltration. However, the causal relationship between them and the specific regulatory mechanisms are unknown. A large number of future experimental studies are needed for verification.

We performed functional enrichment analysis to further explore the possible biological functions played by the DEGs identified in different ARG patterns, GO and KEGG enrichment analyses were performed. These genes mainly enrich biological processes and molecular functions. More precisely, enrichment was most significant in regulating the phagocytosis process and cellular components in the lumen or membrane of specific or tertiary granules. Through a multi-granule delivery system, neutrophils release a wide range of antibacterial compounds and damaging enzymes [32]. AMI leads to a large number of apoptotic cells, and macrophages, on the one hand, promptly remove dying cells and inhibit the release of contents from them, causing secondary necrosis; on the other hand, they can change from a pro-inflammatory phenotype to a reparative phenotype by adjusting gene expression and participate in cardiac remodeling by secreting cytokines, regulating the phagocytic process of macrophages suggests an attractive idea for the treatment of AMI [30]. For instance, the alarming family member S100A9 has been regarded as a potential diagnostic and therapeutic biomarker; long-term blockade will impair the phenotypic switching of reparatory macrophages [36]. In the KEGG analysis, cytokine-cytokine receptor interaction and drug metabolism-cytochrome P450 attracted more attention. Traumatic myocardium triggers oxidative stress, inflammation, and the release of numerous cytokines. Many drugs like taraxerol [37] and gentiopicroside [38], which may reduce inflammatory cytokines, were considered to play a role in lowering myocardial damage. Cytochrome P450 is one of the key monooxygenases in drug metabolism, which can convert arachidonic acid into alcohols and epoxides with anti-inflammatory, antioxidant, and vasodilatory effects, providing myocardial protection after AMI [39].

The network of miRNA and transcription factors displayed potential biomarkers and therapeutic targets for AMI. Several studies have explored the relationship between miRNAs and ARGs in various carcinomas. For example, Zhu et al. found that lncRNA RP11-805J14.5 can compete with CCND2 for miR-34b-3p and miR-139-5p in lung adenocarcinoma [40]. Wen et al. suggested that regulating the TTTY15/miR-98-5p/CCND2 axis may inhibit the progression of gastric cancer [41]. Salem et al. reported that miR-590-3p contributes to ovarian cancer growth and metastasis by stimulating the FOXA2 vesicant pathway [42]. However, the AMI-related literature is relatively sparse, with a portion of studies on extracellular vesicles (EVs). EVs are membrane vesicles containing lipids, proteins, nucleic acids, and metabolites secreted by cells, which can include three categories: exosomes, microvesicles, and apoptotic bodies. They participate in cellular communication and are widely applied in cardiovascular therapy [30]. Li et al. highlighted that small EVs containing miR-486-5p can promote cardiac angiogenesis through fibroblast MMP19/VEGFA cleavage signaling [43]. Yu et al. revealed that EVs produced from atorvastatin-pretreated mesenchymal stem cells improve heart repair after AMI by altering macrophage polarization through the miR-139-3p/STAT1 pathway [44]. These results indicated that EVs might be a promising treatment for AMI, and more research in this area is still needed. Finally, we also visualized possible gene-targeted drugs, providing a theoretical basis for future research on AMI therapy.

This study has some limitations. First, all sample data were downloaded from the GEO database and did not combine multiple databases constrained by limited public data for a more comprehensive validation. In addition, the AUC of the current model has a significant difference between the training and testing sets, suggesting a possible overfitting of the model. It would require more future data to evaluate the current model's accuracy. Second, the current model lacks several clinical features, which are limited by the available public data. More clinical variables should be considered and introduced into the model to improve its diagnostic efficacy further. Third, the exact regulatory relationships involved in miRNA, TFs, and drugs targeting the signature genes predicted by the database still need to be further explored experimentally. Finally, the assessment of ARGs is based exclusively on the levels of mRNAs reported in the databases described above, which do not reflect the levels of functional proteins. The exact mechanism by which these characterized genes recruit specific immune cells to the myocardial infarct zone and other immune-related processes remains unclear. Their contribution to the progression of AMI requires further experimental validation. Despite some limitations of this study, it provides clues to investigate the potential mechanism of action of ARGs in AMI and offers options for immunotherapy and potential diagnostic markers.

## CONCLUSIONS

In conclusion, we identified seven signature ARGs based on the RF algorithm and constructed a risk predictive model of AMI. Besides, this study splits AMI samples into two clusters depending on their different ARG patterns, and the high expression pattern of ARG may have a higher immunotherapy value. Based on these findings, characterizing the angiogenic features of patients with AMI could help identify populations with specific molecular profiles and provide precise treatments. However, additional multi-omics studies are still needed to further explore the angiogenic profile of AMI and provide new strategies for diagnosis, classification, targeted therapy, and prognosis prediction.

## Supplementary Material

Supplementary Table 1
