# Supplementary material for: Application of angiogenesis-related genes associated with immune infiltration in the molecular typing and diagnosis of acute myocardial infarction
Source: Aging (Albany NY). 2024 Jun 14;16(12):10402–23. doi: 10.18632/aging.205936 (PMC11236325; doi:10.18632/aging.205936)
Supplement: Supplementary Table 1 [file aging-16-205936-s001.pdf]

SUPPLEMENTARY TABLE

Supplementary Table 1. Regulatory network of 7 signature ARGs.

Gene	Transcription factor	miRNA	Drug
PGLYRP1	CEBPB, FOXC1, MEF2A, PPARG, E2F1, E2F6, TP53, and IRF2	hsa-mir-16-5p and hsa-mir-335-5p	
POSTN	JUND, FOXL1, YY1, NFYA, and SRF		
THBD	CREB1, E2F1, E2F6, TP53, MAX, USF1, USF2, SREBF1, ELK1, SREBF2, and EN1,	hsa-mir-192-5p, hsa-mir-215-5p, hsa-mir-335-5p, hsa-mir-578, hsa-mir-1207-5p, hsa-mir-2115-5p, hsa-mir-3127-5p, hsa-mir-4763-3p, hsa-mir-4436b-3p, hsa-mir-4632-5p, hsa-mir-6132, hsa-mir-6735-5p, hsa-mir-6836-5p, hsa-mir-6879-5p, and hsa-mir-7843-5p	CILOSTAZOL, CURCUMIN, LEVOTHYROXINE, SIMVASTATIN, and WARFARIN
JAG1	FOXC1, TFAP2C, TP63, and SOX10	hsa-let-7b-5p, hsa-mir-21-5p, hsa-mir-26a-5p, hsa-mir-26b-5p, hsa-mir-98-5p, hsa-mir-199a-5p, hsa-mir-34a-5p, hsa-mir-199b-5p, hsa-mir-214-3p, hsa-mir-124-3p, hsa-mir-137, hsa-mir-143-3p, hsa-mir-145-5p, hsa-mir-200c-3p, hsa-mir-375, hsa-mir-410-3p, hsa-mir-524-5p, hsa-mir-607, hsa-mir-192-3p, hsa-mir-34b-3p, hsa-mir-1305, hsa-mir-4282, hsa-mir-3613-3p, hsa-mir-4480, hsa-mir-4698, hsa-mir-5003-3p, and hsa-mir-8063	HYDROCORTISONE
VEGFA	GATA2, NFYA, E2F1, TFAP2A, E2F6, TFAP2C, ELK4, RUNX2, EGR1, and EN1	hsa-mir-15a-5p, hsa-mir-16-5p, hsa-mir-17-5p, hsa-mir-20a-5p, hsa-mir-21-5p, hsa-mir-29a-3p, hsa-mir-93-5p, hsa-mir-101-3p, hsa-mir-29b-3p, hsa-mir-106a-5p, hsa-mir-107, hsa-mir-199a-5p, hsa-mir-199a-3p, hsa-mir-147a, hsa-mir-34a-5p, hsa-mir-181a-5p, hsa-mir-203a-3p, hsa-mir-205-5p, hsa-mir-200b-3p, hsa-mir-1-3p, hsa-mir-15b-5p, hsa-mir-133a-3p, hsa-mir-140-5p, hsa-mir-145-5p, hsa-mir-9-5p, hsa-mir-125a-5p, hsa-mir-126-5p, hsa-mir-126-3p, hsa-mir-134-5p, hsa-mir-150-5p, hsa-mir-185-5p, hsa-mir-186-5p, hsa-mir-195-5p, hsa-mir-206, hsa-mir-320a, hsa-mir-200c-3p, hsa-mir-106b-5p, hsa-mir-29c-3p, hsa-mir-299-3p, hsa-mir-296-5p, hsa-mir-361-5p, hsa-mir-302d-3p, hsa-mir-369-3p, hsa-mir-372-3p, hsa-mir-373-3p, hsa-mir-374a-5p, hsa-mir-378a-3p, hsa-mir-383-5p, hsa-mir-330-3p, hsa-mir-335-5p, hsa-mir-423-3p, hsa-mir-424-5p, hsa-mir-20b-5p, hsa-mir-429, hsa-mir-329-3p, hsa-mir-410-3p, hsa-mir-495-3p, hsa-mir-497-5p, hsa-mir-520g-3p, hsa-mir-520h, hsa-mir-503-5p, hsa-mir-504-5p, hsa-mir-567, hsa-mir-568, hsa-mir-576-5p, hsa-mir-603, hsa-mir-646, hsa-mir-363-5p, hsa-mir-297, hsa-mir-16-1-3p, hsa-mir-101-5p, hsa-mir-7-1-3p, hsa-mir-7-2-3p, hsa-mir-141-5p, hsa-mir-125a-3p, hsa-mir-34b-3p, hsa-mir-362-3p, hsa-mir-374b-5p, hsa-mir-374b-3p, hsa-mir-1293, hsa-mir-205-3p, hsa-mir-670-5p, hsa-mir-718, hsa-mir-3126-5p, hsa-mir-3163, hsa-mir-4263, hsa-mir-3646, hsa-mir-3662, hsa-mir-3924, hsa-mir-3941, hsa-mir-4483, hsa-mir-4497, hsa-mir-4524a-5p, hsa-mir-4719, hsa-mir-451b, hsa-mir-4735-5p, hsa-mir-4789-5p, hsa-mir-5193, hsa-mir-4524b-5p, hsa-mir-5682, hsa-mir-5692c, hsa-mir-5688, hsa-mir-5692a, hsa-mir-5694, hsa-mir-5692b, hsa-mir-660-3p, hsa-mir-1277-5p, hsa-mir-95-5p, hsa-mir-598-5p, hsa-mir-942-3p, hsa-mir-1252-3p, hsa-mir-6745, hsa-mir-6748-5p, hsa-mir-6756-5p, hsa-mir-6759-5p, hsa-mir-6766-5p, hsa-mir-6769a-5p, hsa-mir-6793-5p, hsa-mir-6838-5p, hsa-mir-6769b-5p, hsa-mir-6870-3p, hsa-mir-6871-3p, hsa-mir-6873-5p, hsa-mir-6875-5p, hsa-mir-1-5p, and hsa-mir-8485	AFLIBERCEPT, ZALTRAP, MUPARFOSTAT, CONBERCEPT, BROLUCIZUMAB, BEVACIZUMAB, RANIBIZUMAB, PEGAPTANIB SODIUM, REGORAFENIB, NAVICIXIZUMAB, LENALIDOMIDE, FLUOROURACIL, BEVASIRANIB, SORAFENIB, BEVACIZUMAB 111IN, LEUCOVORIN, CDC-801, IRINOTECAN, ABICIPAR PEGOL, RISUTEGANIB, CISPLATIN, OSI-632, SILDENAFIL, OXALIPLATIN, DOCETAXEL, FENOFIBRATE, SUNITINIB, CELECOXIB, GENTAMICIN, MP-0250, CARBOPLATIN, DOMATINOSTAT, CAPECITABINE, SQUALAMINE, CILOSTAZOL, ENALAPRIL, PHENYTOIN, and ELMIRON

VCAN	GATA2, FOXL1, NFYA, HNF4A, NFKB1, RELA, TP53, and STAT1	hsa-mir-103a-3p, hsa-mir-107, hsa-mir-129-5p, hsa-mir-218-5p, hsa-mir-23b-3p, hsa-mir-302c-5p, hsa-mir-507, hsa-mir-545-3p, hsa-mir-557, hsa-mir-578, hsa-mir-643, hsa-mir-361-3p, hsa-mir-335-3p, hsa-mir-450b-5p, hsa-mir-3185, hsa-mir-3680-3p, hsa-mir-4436b-5p, hsa-mir-5003-3p, hsa-mir-5011-3p, hsa-mir-5197-3p, hsa-mir-4666b, hsa-mir-552-5p, hsa-mir-627-3p, and hsa-mir-6777-3p	CYCLOSPORINE
CCND2	GATA2, POU2F2, E2F1, HINFP, TFAP2A, MAX, USF1, USF2, EN1, and E2F4	hsa-let-7a-5p, hsa-let-7b-5p, hsa-mir-15a-5p, hsa-mir-16-5p, hsa-mir-17-5p, hsa-mir-19a-3p, hsa-mir-19b-3p, hsa-mir-20a-5p, hsa-mir-26a-5p, hsa-mir-26b-5p, hsa-mir-29a-3p, hsa-mir-96-5p, hsa-mir-98-5p, hsa-mir-29b-3p, hsa-mir-196a-5p, hsa-mir-198, hsa-mir-30c-5p, hsa-mir-182-5p, hsa-mir-15b-5p, hsa-mir-124-3p, hsa-mir-130a-3p, hsa-mir-191-5p, hsa-mir-146a-5p, hsa-mir-154-5p, hsa-mir-185-5p, hsa-mir-195-5p, hsa-mir-206, hsa-mir-320a, hsa-mir-155-5p, hsa-mir-106b-5p, hsa-mir-29c-3p, hsa-mir-302a-3p, hsa-mir-301a-3p, hsa-mir-130b-3p, hsa-mir-302b-3p, hsa-mir-302c-3p, hsa-mir-302d-3p, hsa-mir-372-3p, hsa-mir-373-3p, hsa-mir-378a-5p, hsa-mir-378a-3p, hsa-mir-382-5p, hsa-mir-340-3p, hsa-mir-342-3p, hsa-mir-324-3p, hsa-mir-335-5p, hsa-mir-424-5p, hsa-mir-497-5p, hsa-mir-520e, hsa-mir-519c-3p, hsa-mir-520a-3p, hsa-mir-519b-3p, hsa-mir-520b, hsa-mir-520c-3p, hsa-mir-520d-3p, hsa-mir-519a-3p, hsa-mir-503-5p, hsa-mir-505-3p, hsa-mir-603, hsa-mir-610, hsa-mir-615-3p, hsa-mir-548c-3p, hsa-mir-646, hsa-mir-656-3p, hsa-mir-454-3p, hsa-mir-765, hsa-let-7a-3p, hsa-mir-16-1-3p, hsa-mir-33a-3p, hsa-mir-192-3p, hsa-mir-145-3p, hsa-mir-138-1-3p, hsa-mir-340-5p, hsa-mir-423-5p, hsa-mir-545-5p, hsa-mir-890, hsa-mir-541-5p, hsa-mir-744-3p, hsa-mir-877-5p, hsa-mir-877-3p, hsa-mir-665, hsa-mir-301b-3p, hsa-mir-1228-3p, hsa-mir-1237-3p, hsa-mir-1200, hsa-mir-663b, hsa-mir-1297, hsa-mir-1248, hsa-mir-1255a, hsa-mir-1263, hsa-mir-302e, hsa-mir-1252-5p, hsa-mir-1255b-5p, hsa-mir-2114-3p, hsa-mir-2682-3p, hsa-mir-3125, hsa-mir-3134, hsa-mir-3163, hsa-mir-3173-3p, hsa-mir-4295, hsa-mir-4303, hsa-mir-4306, hsa-mir-4275, hsa-mir-3200-5p, hsa-mir-3613-3p, hsa-mir-3616-3p, hsa-mir-3653-3p, hsa-mir-3666, hsa-mir-3672, hsa-mir-3916, hsa-mir-4419a, hsa-mir-4428, hsa-mir-4434, hsa-mir-4436a, hsa-mir-4465, hsa-mir-4503, hsa-mir-2392, hsa-mir-4510, hsa-mir-4516, hsa-mir-4521, hsa-mir-4524a-5p, hsa-mir-4524a-3p, hsa-mir-3074-5p, hsa-mir-3976, hsa-mir-4644, hsa-mir-4668-5p, hsa-mir-4668-3p, hsa-mir-4709-3p, hsa-mir-4779, hsa-mir-4795-5p, hsa-mir-5000-3p, hsa-mir-5192, hsa-mir-4524b-5p, hsa-mir-548aw, hsa-mir-5692c, hsa-mir-5692a, hsa-mir-5697, hsa-mir-5703, hsa-mir-5692b, hsa-mir-548g-5p, hsa-mir-1277-5p, hsa-mir-548x-5p, hsa-mir-548aj-5p, hsa-mir-6127, hsa-mir-6129, hsa-mir-6130, hsa-mir-6133, hsa-mir-6501-5p, hsa-mir-6505-5p, hsa-mir-1468-3p, hsa-mir-548f-5p, hsa-mir-6730-3p, hsa-mir-6744-5p, hsa-mir-6751-3p, hsa-mir-6760-5p, hsa-mir-6765-3p, hsa-mir-6770-5p, hsa-mir-6772-3p, hsa-mir-6781-3p, hsa-mir-6792-5p, hsa-mir-6797-5p, hsa-mir-6823-3p, hsa-mir-6827-3p, hsa-mir-6828-5p, hsa-mir-6838-5p, hsa-mir-6847-5p, hsa-mir-6859-5p, hsa-mir-6864-3p, hsa-mir-6868-3p, hsa-mir-6878-5p, hsa-mir-6891-5p, hsa-mir-7152-5p, hsa-mir-7849-3p, hsa-mir-8063, hsa-mir-8064, hsa-mir-8083, hsa-mir-1199-5p, hsa-mir-203a-5p, and hsa-mir-1249-5p	RIBOCICLIB and ABEMACICLIB
